# Formulation optimization of Docetaxel loaded self-emulsifying drug delivery system to enhance bioavailability and anti-tumor activity

**DOI:** 10.1038/srep26895

**Published:** 2016-05-31

**Authors:** Guru R. Valicherla, Kandarp M. Dave, Anees A. Syed, Mohammed Riyazuddin, Anand P. Gupta, Akhilesh Singh, Wahajuddin, Kalyan Mitra, Dipak Datta, Jiaur R. Gayen

**Affiliations:** 1 Pharmacokinetics and Metabolism Division, CSIR-Central Drug Research Institute, Lucknow, India; 2 Academy of Scientific and Innovative Research, New Delhi, India; 3 Department of Pharmaceutics, National Institute of Pharmaceutical Education and Research, Raibarelly, India; 4 Biochemistry Division, CSIR-Central Drug Research Institute, Lucknow, India; 5 Sophisticated Analytical Instrumental Facility, CSIR-Central Drug Research Institute, Lucknow, India

## Abstract

Poor bioavailability of Docetaxel (DCT) arising due to its low aqueous solubility and permeability limits its clinical utility. The aim of the present study was to develop DCT loaded self-emulsified drug delivery systems (D-SEDDS) and evaluate its potential ability to improve the oral bioavailability and therapeutic efficacy of DCT. D-SEDDS were characterized for their *in vitro* antitumor activity, *in situ* single pass intestinal perfusion (SPIP), bioavailability, chylomicron flow blocking study and bio-distribution profile. The D-SEDDS were prepared using Capryol 90, Vitamin E TPGS, Gelucire 44/14 and Transcutol HP with a ratio of 32.7/29.4/8.3/29.6 using D-Optimal Mixture Design. The solubility of DCT was improved upto 50 mg/mL. The oral bioavailability of the D-SEDDS in rats (21.84 ± 3.12%) was increased by 3.19 fold than orally administered Taxotere (6.85 ± 1.82%). The enhanced bioavailability was probably due to increase in solubility and permeability. In SPIP, effective permeability of D-SEDDS was significantly higher than Taxotere. D-SEDDS showed 25 fold more *in vitro* cytotoxic activity compared to free DCT. Chylomicron flow blocking study and tissue distribution demonstrated the intestinal lymphatic transport of D-SEDDS and higher retention in tumor than Taxotere. The data suggests that D-SEDDS showed desired stability, enhanced oral bioavailability and *in vitro* antitumor efficacy.

Docetaxel (DCT), a second generation taxoid, is twice as potent as Paclitaxel (PCT) in stabilization and inhibition of microtubule depolymerisation *in vitro*^1^. It is the licensed drug for the treatment of breast cancer with a first-line chemotherapy regimen^2^. The injectable marketed product Taxotere, in which DCT was solubilised in a Tween 80 and 13% ethanol solution, has been reported to cause several side effects due to both drug and solvent system^3^. Similar side effects have been observed for another marketed product, Duopafei^4^. At present, Taxotere and Duopafei are available for clinical use but still oral administration is restricted due to poor aqueous solubility, P-glycoprotein (P-gp) mediated drug efflux, pre-absorptive metabolism by gut membrane-bound cytochrome enzymes, hepatic first-pass metabolism, and decreased gastrointestinal membrane permeability that leads to low oral bioavailability^5^,^6^,^7^.

Lipid-based self-emulsifying drug delivery systems (SEDDS) have attracted the attention of several research groups, due to their potential ability to enhance the oral bioavailability of DCT. SEDDS can enhance the bioavailability by avoiding the first pass metabolism, increasing drug stability in harsh GI environment, facilitating intestinal lymphatic transport of drugs, preventing pre-absorptive metabolism by gut membrane-bound cytochrome enzymes and inhibiting P-gp mediated drug efflux^8^,^9^. Several commonly used excipients in SEDDS such as Cremophor, Tween 80, Labrasol and Transcutol HP could inhibit the function of P-gp mediated drug efflux^6^,^9^,^10^,^11^. Capryol 90 (Polyethylene glycol monocaprylate) and Transcutol (Diethylene glycol monoethyl ether) lead to better solubilization of DCT compared to other lipids and co-emulsifiers^6^,^12^. Gelucire 44/14 (PEG-32 lauric glyceride, HLB 14) and Vitamin E TPGS (Vit E TPGS) (α-Tocopherol PEG 1000 succinate) have been shown to cause highest P-gp efflux activity among all emulsifiers^13^,^14^,^15^.

For optimizing the SEDDS formulation, the relationship between formulation variables and quality attributes must be understood. Design of Experiment (DoE) is a systematic approach for simultaneous evaluation of variables (process or formulation) to develop a product with the desired quality attributes^16^,^17^. It is an efficient method to understand the relationship between the independent and dependent variables^18^. The applicability of DoE in pharmaceutical industries is increasing enormously because FDA recommends and approves quality by design based experiments^19^.

In this present study, DCT-loaded SEDDS (D-SEDDS) were formulated to improve the oral bioavailability as well as antitumor properties of DCT. Capryol 90, Transcutol HP, Gelucire 44/14 and Vit E TPGS were used to prepare the D-SEDDS. Characterization of optimized D-SEDDS included solubility analysis, droplet size analysis by using Zeta Sizer, Transmission Electron Microscopy, stability at different dilutions, freeze thaw stability and cumulative *in vitro* drug release. *In vitro* antitumor activity in MCF-7 cells, *in situ* single pass intestinal perfusion (SPIP), bioavailability and chylomicron flow blocking study were also performed. In addition, bio-distribution of DCT in tissues upon administration of optimized formulation was compared with commercially available Taxotere.

## Results

### Solubility of DCT in different excipients

To increase the solubility of DCT in SEDDS, solubility studies were performed to select the excipients with higher solubilization capacity. Maximum solubility of DCT was observed in Transcutol HP (173.13 ± 5.96 mg/mL) followed by Gelucire 44/14 (54.39 ± 1.87 mg/mL), Vit E TPGS (47.63 ± 1.56 mg/mL) and Capryol 90 (45.15 ± 6.03 mg/mL). Based on saturation solubility studies, these excipients were chosen for further studies.

### Optimization of variables for preparation of D-SEDDS

Before approaching DoE, preliminary formulations were developed and evaluated. Visual appearance suggested that a surfactant concentration of greater than 60% solidified the formulations while a Capryol 90 concentration of greater than 40% decreased the solubility and increased the droplet size. Though possessing higher solubility in Transcutol HP, a Transcutol HP concentration of greater than 30% increased the droplet size with undesired distribution (polydispersity index, PDI > 4).

Response data for all experimental runs of D-Optimal mixture design is summarized in Table 1. Analysis of variance (ANOVA) was applied to determine and understand the significance of the effects of each variable and their interactions. As shown in Supplementary Table S1, ANOVA results suggested linear model as the best fit model for both responses.

### Influence of formulation variables on response Y1 (solubility of DCT)

**Figure 1:**
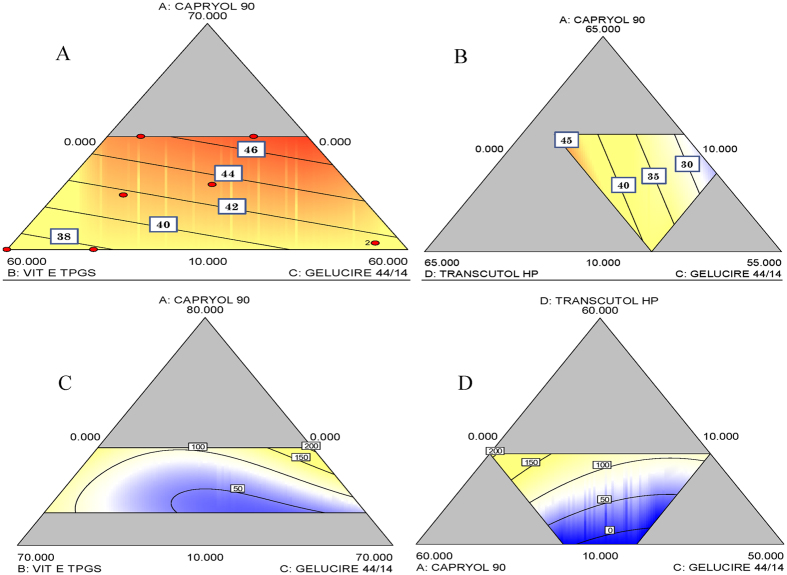
Diagrammatic representation showing the effect of concentrations of formulation variables Capryol 90 (X1), Vit E TPGS (X2), Gelucire 44/14 (X3) and Transcutol HP (X4) on response Y1 (**A,B**), solubility of DCT and response Y2 (**C,D**), post dilution droplet size.

In polynomial regression equation, positive sign indicates a synergistic effect and negative sign indicates an antagonistic effect. In the linear model of response Y1, all four factors (Capryol 90, Vit E TPGS, Gelucire 44/14 and Transcutol HP) had the positive effect in which Vit E TPGS had significantly low effect. As the concentration of Capryol 90 increased from 10 to 40% w/w, the solubility of DCT was increased from 40 to 49 mg/mL, while as the concentration of Gelucire 44/14 increased from 0 to 60% w/w, the solubility of DCT was increased from 38 to 46 mg/mL at highest concentration of Transcutol HP screened which was 30% w/w. However, increasing the concentration of Vit E TPGS (0–60% w/w) decreased the solubility of DCT from 46 to 38 mg/mL showed in Fig 1A. As shown in Fig. 1B, the solubility of DCT was increased from 30 to 45 mg/mL as the concentration of Transcutol HP increases.

### Influence of formulation variables on response Y2 (droplet size upon dilution)

In linear model of response Y2 except Gelucire 44/14, all factors showed positive effects on droplet size upon dilution. i.e., as concentration increased, droplet size was increased. Figure 1C,D shows the correlation between excipients on droplet size. As the concentration of Capryol 90 increased from 10–40% w/w, size was increased from 50 to 100 nm while upon increasing the concentration of Gelucire 44/14 from 0–60% w/w, droplet size was decreased from 200 to 50 nm at concentration of Transcutol HP (20% w/w) (Fig. 1C). As shown in Fig. 1D, keeping the optimum concentration of Vit E TPGS (30% w/w), as the concentration of Gelucire 44/14 increased from 0 to 60% w/w, size was significantly decreased from 200 to 50 nm while increasing the concentration of Capryol 90 and Transcutol HP increased the droplet size.

**Figure 2:**
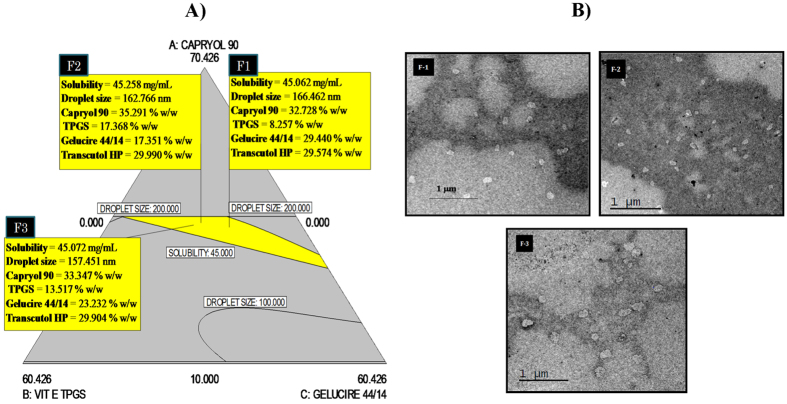
(**A**) Desirable design space (yellow region) based on the effect of formulation variables on the responses, solubility of DCT and post dilution droplet size. (**B**) Transmission electron microphotography images of D-SEDDS: F-1, F-2, and F-3. Average particle size 125 ± 10 nm.

Design space (DS) is the combination and interaction of independent variables to provide quality assurance and is determined from the common region of successful operating ranges for the two responses. For D-SEDDS, levels of factors which provided DCT solubility (Y1) in 45–50 mg/mL range and droplet size (Y2) in 100–200 nm range were screened from which three formulations were selected. These are shown in Fig. 2A as flag (F1, F2 and F3) with predicted DCT solubility and post dilution droplet size. These three selected D-SEDDS were formulated and compared with predicted values of solubility and droplet size as suggested by the linear model.

### Characterization of D-SEDDS

D-SEDDS was characterized for various quality attributes. The average droplet size and solubility of optimized D-SEDDS were compared experimentally with predicted values by DS. As shown in Table 2, solubility of DCT in D-SEDDS (48.35 ± 1.064, 47.90 ± 0.299 and 44.32 ± 0.923 mg/mL in F-1, F-2 and F-3, respectively) was nearer to predicted values (no significant difference). Among the three D-SEDDS prepared, highest DCT solubility was recorded in F-1 (48.35 ± 1.06 mg/mL). Capryol 90 which was used in higher concentration in F-1 amongst the three formulations probably led to the improved solubility while the droplet size of all three formulation were around 160–180 nm (Zeta Sizer) which was consistent with Transmission Electron Microscopy (TEM) results (140–160 nm) shown in Fig. 2B.

Supplementary Table S2 shows the droplet size of D-SEDDS at 2 h after dilution (200, 400, 600 and 800 fold) with SGF (pH 1.2). The results suggested that the submicron emulsions formed after dilutions were to be stable in all the cases without any phase separation. The freeze thaw (freezing at −20 °C for 24 h and thawing at 25 °C for 24 h) stability after 3 consecutive freeze thaw cycles (n = 3) shown in Supplementary Table S3, suggested a marginal change in the droplet size of emulsions (F-2 and F-3) after the second and third freeze thaw cycles. However the values were almost same for F-1 in all three cycles. Similar to the droplet size, there was no significant difference observed in drug content of F-1. Drug precipitation was observed in F-2 and F-3. F-1 was the most stable formulation under freeze thaw condition and it was used for the further remaining *in vitro* and *in vivo* experiments.

### *In vitro* DCT release from D-SEDDS

**Figure 3:**
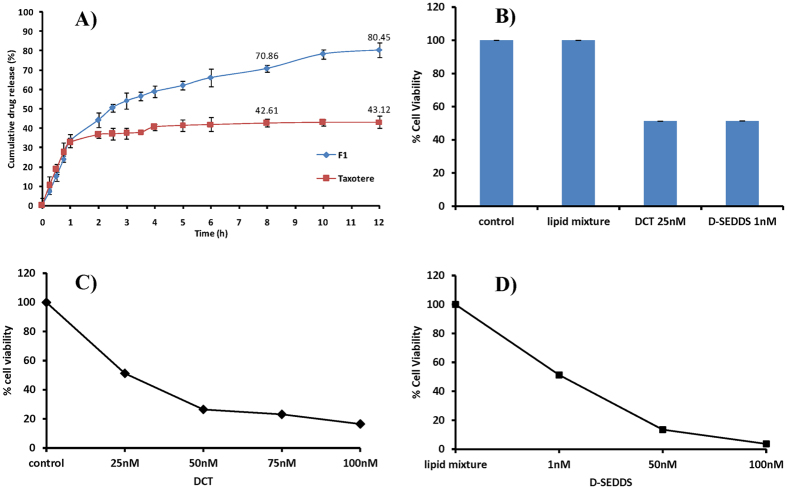
Representation of cumulative *in vitro* drug release and *in vitro* cytotoxicity studies. (**A**) Cumulative drug release profiles of D-SEDDS and Taxotere in SGF/SIF media containing 0.1% Tween 80 by dialysis bag method at 37 °C. Each data point represents the mean ± SD (n = 3). (**B**) Percentage cell viability observed after 72 h treatment of free DCT and D-SEDDS in MCF-7 cells. Percentage cell viability observed after 72 h treatment at different concentrations of (**C**) free DCT and (**D**) D-SEDDS. The results are expressed as mean ± standard deviation (n = 6).

The *in vitro* release profile of DCT from F-1 is shown in Fig. 3A. The *in vitro* cumulative release of DCT from Taxotere and D-SEDDS were found to be 43.12 ± 3.16% and 80.45 ± 3.71%, respectively. Dialysis studies showed that about 80% of drug was released from D-SEDDS within 12 h through a 12 kD membrane and no initial burst release was observed.

### *In vitro* cytotoxic activity

In the MCF-7 cell line, the IC_50_ values of free DCT and D-SEDDS were found to be 25 nM and 1 nM, respectively (Fig. 3B–D). SEDDS (lipid mixture) without DCT was taken as a control where cell viability was 100%. Improved cytotoxic activity of DCT was observed with D-SEDDS. D-SEDDS (F-1) showed 25 fold more *in vitro* cytotoxicity compared to the free drug.

### *In situ* single pass intestinal perfusion (SPIP)

An *in situ* intestinal perfusion study of Taxotere and D-SEDDS was carried out in male Sprague Dawley (SD) rats. The effective permeabilities (*P*_eff_) of Taxotere and D-SEDDS were observed to be 1.04 ± 0.02 × 10^−4^ cm/sec and 5.71 ± 0.24 × 10^−4^ cm/sec (P < 0.05), respectively. There was a significant increase in *P*_eff_ for D-SEDDS as compared to Taxotere.

### *In vivo* pharmacokinetic (PK) studies

**Figure 4:**
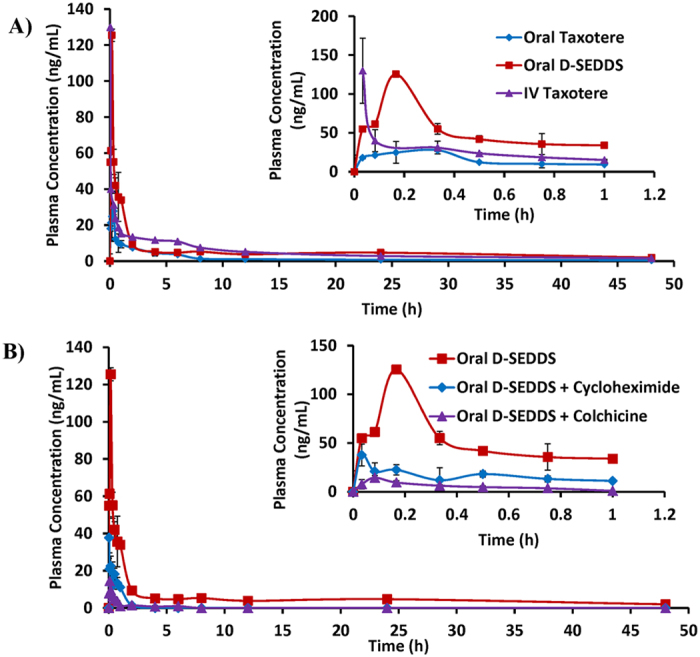
(**A**) Plasma concentration-time profile of DCT after i.v administration of Taxotere (2 mg/kg, equivalent to free DCT), p.o administration of Taxotere (10 mg/kg, equivalent to free DCT) and D-SEDDS (10 mg/kg, equivalent to free DCT). (**B**) Plasma concentration-time profile of DCT after p.o administration of D-SEDDS (10 mg/kg, equivalent to free DCT), Cycloheximide treated D-SEDDS (10 mg/kg, equivalent to free DCT) and Colchicine treated D-SEDDS (10 mg/kg, equivalent to free DCT). Insight shows the expanded plasma concentration profile for 0–1 h duration. The data are presented as mean ± SEM (n = 6/group).

The plasma concentration–time profiles of DCT following 2 mg/kg (equivalent of free DCT) of i.v Taxotere, 10 mg/kg (equivalent to free DCT) of oral Taxotere and equivalent dose of oral D-SEDDS are shown in Fig. 4A. The C_max_ of i.v Taxotere, oral Taxotere and oral D-SEDDS were found to be 129.9 ± 41.72, 35.2 ± 9.70 and 125.5 ± 2.5 ng/mL, respectively. In contrast, D-SEDDS showed significantly higher drug plasma levels throughout the study. The corresponding PK parameters calculated using a non-compartment model are listed in Table 3. Oral D-SEDDS led to a 3.57-fold and 3.19-fold increase in C_max_ and AUC_0-∞_ respectively as compared to oral Taxotere. Importantly, it also showed a 3.19 fold higher absolute bioavailability (21.84 ± 3.12%) than Taxotere (6.85 ± 1.82%). Increased mean resident time and decreased clearance suggested that the D-SEDDS can increase the *in vivo* retention time of DCT and distribute it in different tissues.

### Chylomicron flow blocking experiment

In this experiment, cycloheximide and colchicine treated rat models were used to evaluate the intestinal transport of D-SEDDS after oral administration. After i.p treatment of cycloheximide and colchicine, the C_max_ of D-SEDDS was decreased from 125.5 ± 2.5 to 40.93 ± 7.00 ng/mL and 14.44 ± 4.72 ng/mL, respectively (Fig. 4B). Moreover, the AUC_0-∞_ was reduced from 260.23 ± 51.8 to 23.39 ± 5.36 h.ng/mL and 11.29 ± 1.03 h.ng/mL after the cycloheximide and colchicine pretreatment, respectively (Table 3).

### Bio-distribution studies

Tissue distribution studies in tumor mice model showed that D-SEDDS exhibited a significantly (p < 0.01) higher AUC_0-∞_ (3.00 fold) than Taxotere in tumor tissue. Interestingly, the tumor concentration-time profile clearly suggested that DCT from D-SEDDS showed higher and longer retention in breast tumor at each study point upto 72 h while after 48 h DCT from the Taxotere was completely eliminated from tumor.

**Figure 5:**
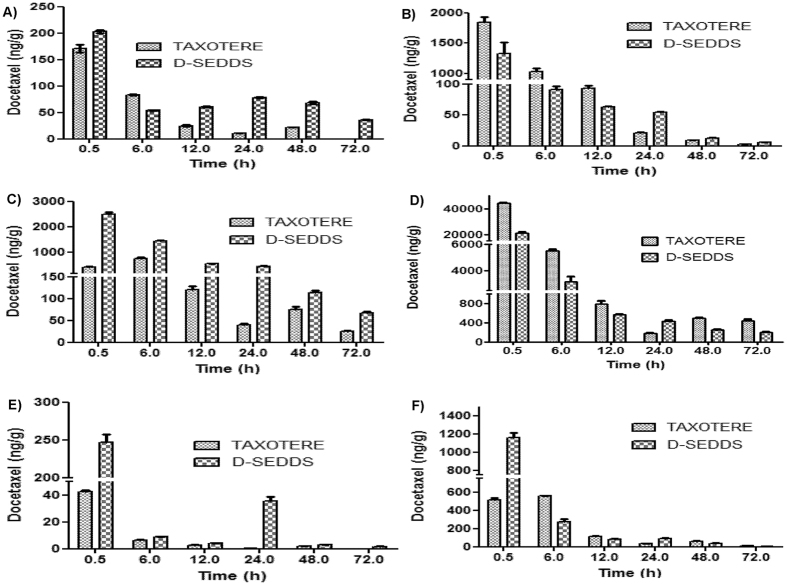
Bio-distribution of Taxotere and D-SEDDS at different time points 30 min, 6 h, 12 h, 24 h, 48 h and 72 h in different tissues of (**A**) Tumor tissue of female breast cancer mice. (**B**) Liver, (**C**) Spleen, (**D**) Intestine, (**E**) Brain and (**F**) Kidney tissues of female SD rats. Data are presented as mean ± SEM (n = 3).

SEDDS enhances the oral bioavailability of BCS class IV drugs by lymphatic transport and decreasing first pass metabolism through liver. To confirm the intestinal lymphatic transport of D-SEDDS, tissue distribution studies in SD female rats were performed after the oral administration of 20 mg/kg Taxotere and D-SEDDS (Fig. 5). Looking at PK parameters of the liver, the higher AUC_0-∞_ of Taxotere than D-SEDDS suggested the hepatic transport of Taxotere a probable reason for low bioavailability. In conjunction with these results, the higher AUC_0-∞_ of D-SEDDS in the spleen than Taxotere suggested the lymphatic transport of DCT from D-SEDDS. In addition, higher levels of DCT in the intestine upon Taxotere administration than D-SEDDS indicate higher absorption of DCT by D-SEDDS. As kidney and brain tissues express high levels of P-gp, the relatively higher concentration of DCT observed from D-SEDDS compared to Taxotere was probably due to P-gp the inhibition effect of D-SEDDS.

## Discussion

For the development of SEDDS, solubility assessment of DCT in suitable oils and surfactants is a crucial step because it defines the drug loading and increases the chylomicron mediated transport of lipophilic drug via the intestinal lymphatic system. Solubility screening of DCT has been reported with several excipients, of which Capryol 90 and Transcutol HP showed the highest solubility^6^,^12^. In our study, DCT also showed higher solubility in Transcutol HP and Capryol 90, thus these were selected as appropriate co-surfactants and oil, respectively for the development of D-SEDDS. Gelucire 44/14 and Vit E TPGS showed the highest P-gp efflux activity and were selected as surfactants.

After extensive optimization, D-SEDDS showed mono-dispersed droplets with a size <200 nm. After oral administration, SEDDS have to encounter various degrees of dilutions in different parts of GIT. Hence the prepared D-SEDDS were evaluated for signs of phase separation after various degrees of dilutions. From Supplementary Table S2, the developed D-SEDDS was found to be stable without any phase separation under all dilution factors. The freeze thaw stability was also evaluated and D-SEDDS showed stable characteristics of droplet size and drug content (Supplementary Table S3). The emulsification capacity of emulsifiers is reduced upon dilution and so at higher concentrations of emulsifiers, drug may precipitate out upon dilution. In Taxotere, DCT is solubilized in Tween 80 and ethanol so upon dilution these surfactants and co-solvents decrease their emulsification and solubilizing capacity causing the drug to precipitate.

The *in vitro* release study was performed in SGF (pH 1.2) for 2 h and SIF (pH 6.8) for 6 h using a dialysis membrane of 12 kD. D-SEDDS (80.45 ± 3.71%) showed more cumulative *in vitro* release compared to Taxotere (43.12 ± 3.16%).

D-SEDDS showed enhanced antitumor activity in the *in vitro* cytotoxicity assay using the MCF-7 cell line. D-SEDDS (F1) showed 25 fold more *in vitro* cytotoxicity compared to the free drug. Multi drug resistance protein like breast cancer receptor proteins (BCRP) which are expressed in MCF-7 cells, might be inhibited by Gelucire 44/14 and Vit E TPGS. The free drug was effluxed by the BCRP proteins in cells but D-SEDDS effectively entered into the cells and showed improved cytotoxic activity.

From the results of the SPIP studies, a marked increase was observed in *P*_eff_ of D-SEDDS compared to Taxotere. The increase in permeability of DCT in D-SEDDS could be due to the effect of P-gp inhibitors like Gelucire 44/14 and Vit E TPGS. Gelucire 44/14 and Vit E TPGS inhibit the P-gp efflux and increase the permeability of DCT in D-SEDDS.

Following the i.v administration of Taxotere, the plasma concentration was decreased to below 20 ng/mL within 2 h. In addition, after oral administration of Taxotere, the AUC_0-∞_ (81.55 ± 17.29 h*ng/mL) and C_max_ (35.2 ± 9.7 ng/mL) were found to be very low probably due to the poor absorption. D-SEDDS showed a promising increase in the plasma concentration compared to Taxotere. D-SEDDS improved AUC_0-∞_ (260.23 ± 51.8 h*ng/mL) and C_max_ (125.5 ± 2.5 ng/mL) in comparison to Taxotere. D-SEDDS (21.85%) enhanced the oral bioavailability by 3.19 fold than oral Taxotere. The enhanced oral bioavailability of D-SEDDS could be due to combined effect of improved solubility, inhibition of P-gp efflux and increased intestinal lymphatic transport of drug consequently reducing first pass metabolism.

As reported in literature after oral administration, D-SEDDS were mixed with GI fluids where spontaneous emulsification occurs and produces oily droplets of size <200 nm. Further, lipolyzation of oil droplets produce emulsified diglycerides, monoglycerides and fatty acids. The emulsified components produce mixed micelles in the presence of bile acids. These mixed micelles are absorbed by the enterocytes where chylomicrons formation occurs. Chylomicrons along with the drug are transported into lymphatic vessel, thus bypassing the direct drug transport to liver^20^. Thus to investigate the intestinal transport of D-SEDDS after its uptake into the enterocytes, cycloheximide and colchicine were used to inhibit the lymphatic transport pathway of lipid or hydrophobic drugs by inhibiting the secretion of chylomicrons from the enterocytes^21^,^22^. It has been reported that cycloheximide inhibits the lymphatic transport without non-specific damage to other passive and active absorption pathways, while colchicine inhibits the lymphatic transport causing non-specific damage as well and alters other absorption pathways. In chylomicron flow blocking model, C_max_ of D-SEDDS was decreased by 3.06 fold for cycloheximide and 8.69 fold upon colchicine pretreatment. Moreover, AUC_0-∞_ of DCT after D-SEDDS administration was decreased more than 10 fold after the i.p treatment of cycloheximide and colchicine. It has been reported that Vit. E TPGS promotes intestinal lymphatic transport of lipophilic drugs by enhancing the secretion of chylomicrons^23^. The reduced absorption of D-SEDDS in the cycloheximide and colchicine pretreatment resulted due to the blocked intestinal lymphatic pathway. Lipid based formulation like SEDDS and Vit E TPGS improved the intestinal lymphatic transport of DCT in D-SEDDS. It suggests that lymphatic transport pathway played an important role in absorption of D-SEDDS. First pass hepatic metabolism of DCT was reduced by intestinal lymphatic transport.

In tumor tissue, C_max_ of DCT after D-SEDDS (206 ng/mL) administration was found to be higher than Taxotere (178 ng/mL). When Taxotere was administered at 48 h DCT concentration was found to be 21.6 ng/mL and it was not retained in the tumor at 72 h. When D-SEDDS was administered the concentration of DCT at 72 h was 37.1 ng/mL in tumor and it indicated the long lasting retention of DCT which helped to give better pharmacological response when given in chronic dose. The intestinal lymphatics also played an important role in the absorption of products after lipid digestion. Interestingly, tissue distribution studies showed that the D-SEDDS followed lymphatic pathway for drug transport while Taxotere followed hepatic drug transport for systemic availability. The developed D-SEDDS posed potential advantages over the commercial one considering patient compliance and quality of life.

## Conclusion

Lipidic based SEDDS formulations have promising potential in enhancing oral bioavailability of poorly soluble drugs. As an implication of their miniscule globule size and emulsification, the absorption is improved as the micro/nanoemulsified drug can be transported by lymphatic pathway. In present study, Type IIIB LBOFs of DCT was developed using Capryol 90 (oil), Gelucire 44/14, Vit E TPGS (surfactants) and Transcutol HP (co-surfactant) with higher DCT loading (solubility ~50 mg/mL). The formulation was successfully optimized and validated by using Mixture D-optimal design. The oral absorption of DCT in female SD rats was significantly enhanced by D-SEDDS (3.19 fold) compared with Taxotere. The lymphatic pathway of drug transport from D-SEDDS was successfully justified by chylomicron flow blocking model and tissue distribution studies. The effective concentration in solid breast tumors upon single dosing were found to be higher leading to better retention of DCT upto 72 h by D-SEDDS compared to Taxotere in female BALB/c mice. The developed formulation could also be investigated in various different types of cancer. The evaluation of the developed formulation in a suitable *in vivo* cancer model could prove useful to explore the antitumor efficacy of the formulation.

## Materials & Methods

### Chemicals & reagents

DCT was purchased from Swapnroop Drugs Pvt. Ltd., India. Polyglycolyzed glycerides (Capryol 90, Labrasol, Transcutol HP and Gelucire 44/14) were received as generous gifts from Gattefosse, India. Tocopherol polyethylene glycol α succinate (Vit E TPGS) was purchased from Sigma, India. Taxotere was purchased from Sanofi-Aventis. Ethyl acetate and methyl-ter-butyl ether (TBME) were purchased from Spectrochem, India. Methanol and acetonitrile were high performance liquid chromatography (HPLC) and liquid chromatography tandem mass spectrometry (LC-MS/MS) grade and supplied from Sigma, India. All other chemicals and reagents were of analytical or HPLC grade.

### Preparation of D-SEDDS

#### Solubility study

The DCT solubility in different excipients was determined by using shake flask method. An excess amount of DCT (200 mg) was transferred in 5 mL ria vial containing 1 g of selected vehicles, vortexed and operated at 100 strokes/min for 48 h at 37 ± 0.5 °C. Due to semisolid consistency, mixtures of Gelucire 44/14 & Vit E TPGS were incubated in shaker water bath (Julabo SW23) at 60 °C^24^. After 48 h, the mixture was centrifuged at 12000 rpm (Eppendorf, Germany) for 5 min and supernatant was measured by validated HPLC method after proper dilution with mobile phase.

### Analytical method for DCT quantification

Shimadzu UFLC system (Kyoto, Japan) equipped with SPD-M20A PDA detector and SIL-HTc autosampler was used for the analysis of all standards and samples. The compounds were separated on a Phenomenex Luna C18 (150 mm × 4.60 mm, 3 μm) column at 40 °C. The mobile phase was composed of acetonitrile and 10 mM ammonium acetate (47:53, v/v). Efficient and symmetrical peaks were obtained at flow rate of 1 mL/min with a sample injection volume of 50 μL. The detection wavelength was 228 nm for both DCT and PCT.

### Preliminary D-SEDDS preparation

According to lipidic formulation classification system, class IIIA, IIIB & IV emulsions can able to form droplets of 10–200 nm size in which triglycerides should be within the concentration range of 10–50%, while surfactants and co-surfactants should be within the range of 0–80% w/w. To satisfy these conditions preliminary formulation batches were prepared using Capryol 90, Vit E TPGS, Gelucire 44/14 and Transcutol HP in the concentration range of 10–50% w/w of each excipient. Prepared batches were characterized on the basis of visual observation, droplet size and solubility of DCT.

### Design of Experiments (DoE)

DoE was applied for SEDDS formulation with four-factor, two-level D-Optimal mixture statistical design to evaluate the individual and interaction effects of excipients concentration on DCT solubility in D-SEDDS and droplet size of diluted D-SEDDS. The concentration of Capryol 90 (Factor A), Vit E TPGS (Factor B), Gelucire 44/14 (Factor C) and Transcutol HP (Factor D) were varied from 0 to 60%. The effect of these formulation variables (Factors A–D) on dependent variables (Y1: solubility of DCT in SEDDS and Y2: droplet size of diluted D-SEDDS) were studied using Design Expert 8.0.4.1 software. A total of 17 experiments were designed by the software with 2 replicates. To increase the predictability, experiments were performed in random order. A batch size of 100% was kept for all experiments and the combination of Vit E TPGS and Gelucire 44/14 were kept less than or equal to 60% of total weight.

### Characterization of D-SEDDS

#### Average droplet size & polydispersity index

The mean hydrodynamic droplet size and PDI of optimized D-SEDDS were measured at 25 °C by dynamic light scattering using Zeta Sizer (Malvern Instruments, UK) equipped with a He–Ne laser that operated at a wavelength of 635 nm after different dilutions with triple distilled water. The D-SEDDS were transferred to a standard cuvette, and measurements were taken at a fixed scattering angle of 90°. Software was used to analyze the size and PDI. Each sample was analysed in triplicate.

### Transmission Electron Microscopy (TEM)

D-SEDDS were diluted 200 times with triple distilled water and mixed by slightly shaking. One drop of diluted sample was deposited on a carbon-coated copper grid and observed under the TEM.

### Stability studies

#### Stability studies at different dilutions

Stability of D-SEDDS against different dilutions (200, 400, 600 and 800 times) was evaluated with SGF (pH 1.2) and droplet size, of the resultant emulsion were investigated in triplicate^25^.

### Freeze thaw stability

Freeze thaw stability of D-SEDDS was evaluated by exposing to 3 continuous cycles. In each cycle samples, were frozen for atleast 24h at −20 °C followed by thawing at 40 °C for 24 h. The formulations were then evaluated for the droplet size and % drug content in triplicate^25^.

### *In vitro* drug release using dialysis membrane

D-SEDDS and Taxotere (equivalent to 2 mg of DCT) were dispersed in 1 mL of gastrointestinal fluids and filled in preactivated dialysis membrane (12 kD). The drug release was performed using shaking water bath operated at 37 °C at 100 strokes/min in 100 mL of releasing media containing 2.5% w/v Tween 80. *In vitro* release of D-SEDDS and Taxotere were carried out for 2 h in SGF pH 1.2 followed by 10 h in SIF pH 6.8. Samples (1 mL) were taken at the different time intervals and maintained sink condition with 1 mL of fresh medium. The cumulative percentage drug release was analyzed by the validated HPLC method.

### *In vitro* cytotoxicity studies

MCF-7 cells were grown in RPMI 1640 medium (Gibco) containing 10% fatal bovine serum, 50 U/mL penicillin, and 50 mg/mL streptomycin. The cytotoxicity of D-SEDDS was evaluated by MTT assay. Free drug and D-SEDDS were added at equivalent DCT in RPMI 1640 medium. D-SEDDS without DCT was used as control. 5 mg/mL MTT dissolved in PBS and filtered. The cells were treated with the drug at different concentrations for 72 h at 37 °C incubator. 10 μL MTT reagents was added to 100 μL cell culture media and incubated for 3–4 h. After removing the media, 100 μL DMSO was added to dissolve the purple colour formazan crystals. Incubated for 10 min and reading was taken at 570/650 nm. The cell viability (%) was calculated and compared with the untreated control^26^.

### *In situ* single pass intestinal perfusion (SPIP)

The *in situ* SPIP studies of Taxotere and D-SEDDS were carried out in young male SD rats, weighing around 220 ± 20 g, were obtained from the National Laboratory Animal Centre, Central Drug Research Institute (CDRI) (approval number: IAEC/2012/91). Rats were housed in well ventilated cages with standard laboratory conditions with regular 12 h light-dark cycle. The study protocols were approved by the Institutional Animal Ethics Committee, CSIR- Central Drug Research Institute (CDRI), Govt. of India and all the methods were carried out in accordance with the approved guidelines.

Rats were fasted for overnight before the experiment with free access to water and anaesthetized using a urethane injection (1 g/kg, i.p) and kept in a supine position on a heated pad to maintain normal body temperature. A midline longitudinal abdominal incision was carefully made in the abdomen, and the lumen of the jejunum (8–12 cm) was exposed and catheterized with polypropylene perfusion tubing. The drug perfusion solution containing 100 μg/mL DCT (in Taxotere and D-SEDDS) and 100 μg/mL phenol red, was pumped into the intestinal segments at a constant flow rate of 0.2 mL/min. After allowing 30 min to reach steady-state outlet concentrations, outlet perfusate samples were collected every 15 min for 120 min perfusion period. Phenol red was used as a marker of osmosis/zero permeability. At the end, the length of segment was measured without stretching and finally the animal was euthanized. Samples were stored at −20 °C until analysis. The concentration of DCT and phenol red in permeability samples was determined by UFLC method. The phenol red was detected at the wavelength of 420 nm. The SPIP is based on reaching steady state with respect to the diffusion of DCT across intestine. Steady state is confirmed by plotting the ratio of the outlet to inlet concentrations (corrected for water transport) versus time. Permeability calculations across rat jejunum (P_*eff*_) were performed from intestinal perfusate samples collected over 30–120 min (steady state). The effective permeability coefficient (P_*eff*_) and drug absorption rate constant (Ka) were calculated using the following equations:


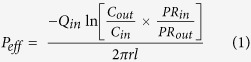



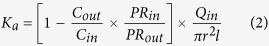


Where, C_in_ and C_out_ are the concentrations of DCT in the entering perfusate and collected perfusate, respectively; PR_in_ and PR_out_ are the concentrations of phenol red in the entering perfusate and collected perfusate, respectively; Q_in_ is the flow rate of entering perfusate; r is the inner radius of the intestine, which is 0.18 cm and l is the length of the intestine (cm)^27^.

### Pharmacokinetic studies

The PK studies of DCT were carried out in young female SD rats. For per-oral (p.o) PK studies, the animals were randomly distributed with proper marking into two groups (D-SEDDS and Taxotere) each containing 6 animals. Taxotere group of animals received oral Taxotere (10 mg/kg, equivalent to free DCT) and D-SEDDS (10 mg/kg, equivalent to free DCT). For intravenous (i.v) PK studies, one group of 6 animals were administered with i.v Taxotere (2 mg/kg, equivalent to free DCT). Under mild anaesthesia of rats, blood samples were collected at predetermined time intervals from the retro-orbital plexus into heparinized microcentrifuge tubes (containing 20 μL of 1000 IU heparin/mL of blood). Blood samples were centrifuged at 5000 rpm for 10 min to separate plasma and kept at −80 ± 10 °C until analysed.

### Chylomicron flow blocking experiment

To investigate the intestinal lymphatic transport of D-SEDDS in rats, chylomicron flow blocking experiment was performed by using cycloheximide and colchicine. Female SD rats (200–220 g) were fasted overnight and the rats were distributed randomly into two groups each containing 6 animals, one group was injected with cycloheximide (3 mg/kg, i.p) dissolved in saline (1.5 mg/mL) and another group injected with colchicine (5 mg/kg, i.p) dissolved in saline (2.5 mg/mL)^21^. After one hour pretreatment, the both groups were orally administered with D-SEDDS (10 mg/kg equivalent to free DCT). Blood samples were collected at predetermined time intervals. The plasma was harvested from blood samples and kept at −80 ± 10 °C until analysed.

#### Liquid chromatography coupled with tandem mass spectrometry (LC-MS/MS) analysis of DCT

Mass spectrometric detection was performed on Qtrap 4000 mass spectrometer (Applied Biosystems, MDS Sciex Toronto, Canada) equipped with an electro-spray ionization source. The analytical column was a Phenomenex Luna C18, (75 mm × 4.60 mm, 3 μ). The mobile phase composition of Methanol: 10 mM Ammonium formate (90:10, v/v) was used. The sample injection volume was 10 μL and the total run time was 3 min using a flow rate of 0.7 mL/min. The transitions (parent to daughter) monitored were m/z 808.4 → 527.2 for DCT and m/z 854.4 → 286.1 for PCT.

### Plasma samples analysis by liquid-liquid extraction (LLE)

To 100 μL of plasma sample, 20 μL of PCT (25 ng/mL) added and vortexed. To that, 100 μL of acetonitrile and 200 μL of water were added and vortexed. In the mixed sample, 2 mL extracting solvent (Ethyl acetate: TBME, 1:1) was added and vortexed for 10 min at 2500 rpm on vibramax (Heidolph, Germany). The mixture was centrifuged at 8000 rpm (Eppendorf, Germany) and supernatant (1.75 mL) was then completely evaporated to dryness in speedvac concentrator (Savant Instrument, Farmingdale, USA). The dried residue was reconstituted with 100 μL mobile phase, after vortexing for 5 min, centrifuged at 12000 rpm for 10 min; a 70 μL aliquot was transferred and injected into the LC-MS/MS system for quantification.

### Distribution of DCT in tumor

To investigate the tumor distribution study of DCT, breast cancer mice model was developed. Female BALB/c mice (6–8 week old, weighing 17–20 g) were injected subcutaneously with 4T1 (murine mammary carcinoma cells) into a mammary gland available in the lower right quadrant of the animal abdomen (1 × 10^6^/mice)^28^. Treatment was initiated when primary tumors reached a mean diameter of 10 mm. For tumor distribution studies, the animals were randomly distributed with proper marking into two groups (D-SEDDS and Taxotere) each containing 18 animals. Taxotere group of animals received oral Taxotere (20 mg/kg, equivalent to free DCT) and D-SEDDS (20 mg/kg, equivalent to free DCT). The animals were sacrificed at periodic intervals (0.5, 6, 12, 24, 48 and 72 h) and tumor tissues were perfused with ice-cold Tris buffer, pH 7.4 and snap-frozen in liquid nitrogen. The frozen samples were stored at −80 °C until further processing. The tumor from control animal (without drug) was also collected and processed the same like treated animals.

### Bio-distribution studies

The female SD rats were randomly distributed with proper marking into two groups (D-SEDDS and Taxotere) each containing 18 animals. Taxotere (20 mg/kg, equivalent to free DCT) and D-SEDDS (20 mg/kg, equivalent to free DCT) were administered orally to each group. The animals were sacrificed at periodic intervals (0.5, 6, 12, 24, 48 and 72 h) and tissues (liver, spleen, intestine, kidney and brain) were collected. The collected tissues were perfused with ice-cold Tris buffer, pH 7.4 and snap-frozen in liquid nitrogen. The frozen samples were stored at −80 °C until further processing. The tissues from control animal (without drug) were also collected and processed the same like treated animals^29^.

The tissue samples were weighed and homogenized in 1:1 ratio with Tris buffer, pH 7.4. The pipettable tissue samples were processed same as plasma samples in pharmacokinetic study mentioned above taking 200 μL of sample volume. The final reconstituted aliquots were analysed by validated LC-MS/MS method.

### Data analysis

Various PK parameters were analysed from bio samples concentration-time profiles, using Phoenix 1.3 (Pharsight, CA, USA). All data were expressed as mean ± standard deviation.

## Additional Information

**How to cite this article**: Valicherla, G. R. *et al.* Formulation optimization of Docetaxel loaded self-emulsifying drug delivery system to enhance bioavailability and anti-tumor activity. *Sci. Rep.* **6**, 26895; doi: 10.1038/srep26895 (2016).

## Supplementary Material

Supplementary Information

## Figures and Tables

**Table 1:** Matrix of 4 factor, 2 level, D-Optimal mixture design and its responses.

Run	A: Capryol 90 (%)	B: Vit E TPGS (%)	C: Gelucire 44/14 (%)	D: Transcutol HP (%)	Y1: Solubility (mg/g)	Y2: Mean droplet size (nm)
A1	10.00	47.01	12.99	30.00	49.19 ± 4.54	189.50
A2	29.60	43.08	16.92	10.40	30.42 ± 4.91	42.70
A3	27.25	20.63	22.12	30.00	45.97 ± 1.52	108.00
A4	33.17	0.00	45.06	21.77	43.99 ± 0.66	134.00
A5	20.78	16.05	43.95	19.22	34.94 ± 1.52	15.99
A6	40.00	24.93	5.07	30.00	46.3 ± 1.97	158.10
A7	24.47	35.31	10.22	30.00	39.26 ± 2.20	70.57
A8	30.00	60.00	0.00	10.00	22.09 ± 1.58	36.19
A9	30.00	0.00	60.00	10.00	27.02 ± 1.70	69.11
A10	38.36	41.36	0.00	20.28	31.52 ± 1.27	189.40
A11	30.00	0.00	60.00	10.00	27.49 ± 2.40	109.90
A12	10.00	60.00	0.00	30.00	30.79 ± 0.72	174.50
A13	21.06	30.87	29.13	18.94	28.7 ± 0.79	15.58
A14	11.71	3.98	54.30	30.00	41.11 ± 5.04	35.24
A15	40.00	20.49	29.51	10.00	29.65 ± 0.37	18.68
A16	40.00	8.04	21.96	30.00	46.22 ± 0.90	274.40
A17	11.71	3.98	54.30	30.00	35.91 ± 0.31	115.20

**Table 2:** Comparison of predicted and experimental values of responses of D-SEDDS.

D-SEDDS	Capryol 90 (%)	Gelucire 44/14 (%)	Vit E TPGS (%)	Transcutol HP (%)	Solubility (mg/g)		PDI	Droplet size (nm)	
					Predicted	Experiment		Predicted	Experiment
F1	32.73	29.44	8.26	29.57	45.06	48.35 ± 1.06	0.11	166.460	167.3 ± 2.30
F2	35.29	17.35	17.37	29.99	45.26	47.90 ± 0.29	0.24	162.760	178.2 ± 1.90
F3	33.35	23.23	13.52	29.90	45.07	44.32 ± 0.92	0.35	157.450	162.6 ± 5.60

Values are presented as mean ± SD (n = 3).

**Table 3:** PK parameters of Taxotere (i.v & p.o), D-SEDDS (p.o), D-SEDDS + Cycloheximide (p.o) and D-SEDDS + Colchicine (p.o).

PK parameters	Units	Intravenous (i.v)	Per oral (p.o)			
		Taxotere	Taxotere	D-SEDDS	D-SEDDS + Cycloheximide	D-SEDDS + Colchicine
Dose	mg/kg	2.00	10.00	10.00	10.00	10.00
T_max_	h	0.03	0.25 ± 0.08	0.17	0.05 ± 0.02	0.083
C_max_	ng/mL	129.9 ± 41.72	35.2 ± 9.70	125.5 ± 2.50	40.93 ± 7.00	14.44 ± 4.72
AUC_0-∞_	h.ng/mL	238.25 ± 4.66	81.55 ± 17.29	260.23 ± 51.8	23.39 ± 5.36	11.29 ± 1.03
CL	L/h/kg	6.61 ± 0.68	98.25 ± 15.79	28.31 ± 3.33	467.82 ± 87.90	900.61 ± 83.19
V_d_	L/Kg	229.73 ± 49.27	4950.92 ± 1725.98	1460.33 ± 484.28	253.41 ± 53.58	2827.60 ± 1376.18
Mean Resident Time	h	13.35 ± 1.98	10.65 ± 0.19	14.90 ± 0.22	0.70 ± 0.03	2.56 ± 0.93
t_1/2_	h	25.30 ± 7.80	33.84 ± 6.70	34.83 ± 7.70	0.38 ± 0.04	2.07 ± 0.85
F	%	–	6.85 ± 1.82	21.84 ± 3.12	–	–

Values are presented as mean ± SEM, n = 6 for i.v and oral.
