# Supplementary material for: Formulation optimization of Docetaxel loaded self-emulsifying drug delivery system to enhance bioavailability and anti-tumor activity
Source: Sci Rep. 2016 May 31;6:26895. doi: 10.1038/srep26895 (PMC4886259; doi:10.1038/srep26895)
Supplement: Supplementary Information [file srep26895-s1.pdf]

Supplementary file

Formulation optimization of Docetaxel loaded self-emulsifying drug delivery system to enhance bioavailability and anti-tumor activity

Guru R.Valicherla[1,5], Kandarp M. Dave[2], Anees A. Syed[1], Mohammed Riyazuddin[1], Anand P. Gupta[1], Akhilesh Singh[3], Wahajuddin[1,5], Kalyan Mitra[4], Dipak Datta[3], Jiaur R. Gayen[1,5,*]

Table S1: Mathematical equation of responses according to D- Optimal Mixture design.

Response	Model	R^2	P value	F value	Reduced regression equation for the response
Solubility	Linear	0.7330	0.0005	11.50	Solubility= 34.62 cap + 6.29 TPGS + 13.84 Gel + 98.51 Trans
Droplet size	Linear	0.7486	0.0088	6.46	Droplet size=521.98 cap+ 128.54 TPGS - 55.91 Gel+ 334.27 Trans- 48.44 cap*TPGS-2910.49 cap*TPGS*Gel

Table S2: Robustness of droplet size after different dilutions. Values are presented as mean± standard deviation (n=3).

Dilutions (fold)	Mean droplet size (nm) ± SD		
	**F-1**	**F-2**	**F-3**
200	167.30±2.30	178.20±1.90	162.60±5.60
400	159.90±1.80	172.30±3.20	160.30±3.20
600	154.20±1.60	170.40±4.30	159.30±4.10
800	146.20±0.34	167.40±1.40	154.20±1.30

Table S3: Measurement of droplet size and drug content after 3 consecutive freeze thaw cycles. Values are presented as mean±standard deviation (n=3).

Cycles	Droplet size (nm)			Drug content (%)		
	**F-1**	**F-2**	**F-3**	**F-1**	**F-2**	**F-3**
Initial	167.30±5.42	177.10±2.18	204.60±3.71	100.00	100.00	100.00
1st	178.10±4.80	338.00±7.21	245.30±10.70	96.66±1.51	95.65±0.54	96.45±1.15
2nd	174.30±8.48	407.33±6.30	344.50±8.73	96.64±0.48	89.85±1.60	90.66±0.53
3rd	181.40±3.74	430.41±4.60	397.80±4.59	93.84±0.54	84.76±0.16	84.31±0.20
